# Organic Light-Emitting Diodes with Electrospun Electrodes for Double-Side Emissions

**DOI:** 10.3390/mi14030543

**Published:** 2023-02-25

**Authors:** Iulia Corina Ciobotaru, Monica Enculescu, Silviu Polosan, Ionut Enculescu, Constantin Claudiu Ciobotaru

**Affiliations:** National Institute of Materials Physics, Laboratory of Functional Nanostructures, Atomistilor 405A, 077125 Magurele, Romania

**Keywords:** transparent conductive electrodes, electrospun web cathodes, electrospinning method, double side-emission

## Abstract

Transparent conductive electrodes (TCE) obtained by the electrospinning method and gold covered were used as cathodes in the organic light-emitting diodes (OLEDs) to create double side-emission. The electro-active nanofibers of poly(methyl methacrylate) (PMMA) with diameters in the range of several hundreds of nanometers, were prepared through the electrospinning method. The nanofibers were coated with gold by sputtering deposition, maintaining optimal transparency and conductivity to increase the electroluminescence on both electrodes. Optical, structural, and electrical measurements of the as-prepared transparent electrodes have shown good transparency and higher electrical conductivity. In this study, two types of OLEDs consisting of indium tin oxide (ITO)/ poly(3,4-ethylenedioxythiophene) polystyrene sulfonate (PEDOT-PSS)/ Ir(III) complex (8-hydroxyquinolinat bis(2-phenylpyridyl) iridium–IrQ(ppy)_2_ 20 wt% embedded in N, N′-Dicarbazolyl-4,4′-biphenyl (CBP) sandwich structure and either gold-covered PMMA electrospun nanoweb (OLED with electrospun cathode) were fabricated together with a similar structure containing thin film gold cathodes (OLED with thin film cathode). The luminance-current-voltage characteristics, the capacitance-voltage, and the electroluminescence properties of these OLEDs were investigated.

## 1. Introduction

The next generation of optoelectronic devices requires industrial-scale and low-cost fabrication methods of OLEDs. The efficiency of these devices can be substantially increased using TCE as the cathode [1,2,3]. The quality and geometry of the cathodes influence the charge injection into the active layer and improve the external quantum efficiency (EQE) [4].

The optical and electrical properties of the TCE are drastically influenced by the quality of the electrodes and the method of deposition. Different parameters like roughness, transparency and work function are crucial for OLED and solar cell devices [5]. Particularly, for OLED devices, a sheet resistance below 10 Ω/sq and an optical transmittance of over 90% is required. These parameters are difficult to achieve in metallic neat films due to the interplay between transparency and electrical conductivity. A thinner film has a higher sheet resistance and a thicker one has a lower transmittance. The change in the metallic thin films with transparent nanofibers provides an opportunity to manipulate the electron injection and the charge transport mechanism in these devices and adjusts the optical transmittance and sheet resistance. Moreover, the light emission through these transparent nanofibers may be driven differently. For the transparent cathodes, obtained from PMMA nanowebs covered with gold thin films and having a thickness between 40 and 50 nm, the contribution of the metal is much larger than that of metal films, avoiding the electron scattering due to the substrate roughness and the strain boundaries decreases, ensuring a better electrical conductivity. For light emission, these nanoweb electrodes create a non-uniform electric field, enhancing the electroluminescence around the nanofibers.

These transparent nanofibers show comparable electrical conductivity and transparency to that of ITO thin films while providing improved flexibility for further foldable OLED devices. However, for these electrospun nanofibers, the wire-to-wire junction resistance must be minimized with the annealing process beyond the tolerance of plastics and a thick planarization of the electrode is needed to decrease the film surface roughness [6,7,8].

The transparent conductive electrodes obtained from the electrospinning method could represent an alternative way to improve the light output of these electroluminescent devices. The PMMA nanofibers may be easily obtained through the electrospinning process, which is now widely used for continuous nanofibers, enabling the control of diameters in the range of several nanometers to the micrometer regime [9]. This method allows the obtaining of continuous nanofibers formed due to the electrostatic Coulombic repulsive forces applied during the elongation of the viscoelastic solution as it strengthens to form a fiber. Various parameters influence the fiber formation, like processing parameters (applied electric field, solution flow rate, diameter of the syringe needle, collector geometry), solution parameters (solution concentration and viscosity, temperature), but also ambient parameters (temperature, humidity) [10,11,12,13,14].

The gold covering of the nanofibers using DC magnetron sputtering allows the obtaining of the transparent and conductive electrodes, which are transferred on the sandwich structure of the OLED. For a double charge injection, the sandwich structure is formed from the glass substrate, covered with a pre-patterned ITO thin film, followed by PEDOT: PSS thin film as the hole transport layer (HTL). The desired organometallic compound 20 wt% dispersed in a transparent/conductive polymer (CBP), forms the emissive layer (EML). The electrospun cathode was thermally transferred over the EML for the electron injection in the OLED structure. The charge injection from the gold cathodes should overcome a higher potential barrier than other metals such as silver and aluminum, to enter into the active layer. This fact results in a poor electron injection requiring a higher drop voltage [15]. However, the use of gold over the nanoweb fibers ensures better adhesion to the OLED organic layer compared to other metals with lower work functions like Ag and Al [16]. This fact can be seen in the SEM images (Figure S3 from supplementary) where the covering process with gold is more adhesive over the OLED structure. The better adhesion of the gold significantly reduces the roughness of the TCE electrodes, which partially compensates for the higher potential barrier and decreases the contact resistance between the metallic electrode and the semiconducting thin films [17]. The magnitude of the potential drop at the contact depends on the Schottky barrier given by the geometry but also by the roughness between the metallic electrode and the semiconductor [18]. Moreover, the gold layer does not have any oxidation problem, compared with silver or aluminum, and is therefore chemically stable in the air [19]. For instance, the air non-stable metals involve supplementary steps like the encapsulation of the final device, which could lead to cathode fiber breakage.

Several TCE electrodes have been proposed for optoelectronic devices where light is extracted from both electrodes, through the anode, usually made from ITO covering a glass substrate, and through cathodes, which are transparent metals with medium transmittance or different metallic configurations such as metal nanogrids, nanowires or nanofibers [5]. The present paper deals with transparent cathodes as nanowebs obtained using the electrospinning method and comprising gold-covered PMMA template nanowebs, which improve the double-side electroluminescence of the OLEDs by increasing their light emission.

The nanowire networks used as electrodes for different optoelectronic devices are made using two routes: bottom-up method, solution process to assemble the nanowires into a network, for which a post-treatment is often required to weld the contacting nanowires; and top-down method, deposition of a metal film onto a template or mask to form a fully inter-connected network without any wire–wire junction resistance. For OLED applications, mainly solution-processed nanowires are employed to obtain TCE [1,8].

In this work, we create cathode nanofibers by electrospinning using the top-down method. The as-prepared transparent PMMA/gold electrodes were thermally attached to the OLED structure to ensure the fusing of the junction between nanowires and to minimize the wire-to-wire junction resistance. The gold-covered PMMA cathodes and the classical thin film cathodes are included to analyze the main properties of the OLED, such as current-voltage measurements and the electroluminescence properties. For the organometallic compound we have used a dual-emitter compound IrQ(ppy)_2_ consisting of the central Ir^3+^, two phenylpyridyne ligands, and one quinoline ligand, which overlaps the green and red electroluminescence [20,21]. The significant advantage of the TCE electrodes obtained using the electrospinning technique is their transparency and conductivity, depending on fiber density, comparable with those of the thin-film electrodes.

## 2. Materials and Methods

### 2.1. Materials

The electrospinning fiber precursor was poly (methyl methacrylate) PMMA (Mw = 350,000, Sigma Aldrich) 10 wt%, dissolved in dimethylformamide (DMF, ≥99.8%, Sigma-Aldrich). The PEDOT-PSS conductive polymer Al 4083, Hellmanex, and pre-patterned ITO substrates were purchased from Ossila. The IrQ(ppy)_2_ emissive organometallic compound was synthesized in our laboratory and described previously [22]. The CBP electrically conductive and transparent material and dichloromethane (DCM) anhydrous were purchased from Sigma-Aldrich. Gold (99.99%) as the sputtering target was purchased from Kurt J. Lesker.

### 2.2. Fabrication of the OLEDs with Electrospun and Thin Film Cathodes

**Figure 1:**
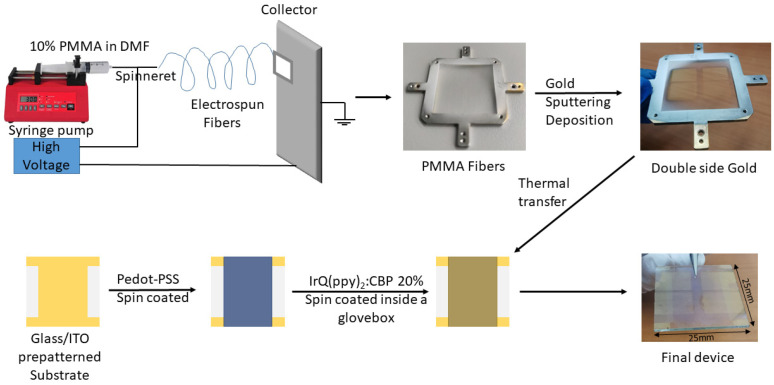
Fabrication procedure of the OLEDs with electrospun cathodes.

The OLED’s fabrication process involves several steps illustrated in Figure 1.

In the first step, the PMMA electro-active nanofibers with diameters in the range of several hundred nanometers were produced using the electrospinning method [23]. The processing parameters are applied electric potential of 15 kV, solution flow rate of 0.5 mL/h, the diameter of the syringe needle of 0.8 mm, and syringe tip-to-collector distance−150 mm. The solution of 10% PMMA in DMF was maintained at 22–24 °C, while the ambient parameters are given by humidity of 27% and the temperature of 22–24 °C. The metallic collector with an inner area of 900 mm^2^ (30 × 30 mm) was made from stainless steel. The PMMA insulator material was used as a template in the design of cathodes obtained after the gold deposition. The gold metal deposition was performed by magnetron sputtering at 18 mA and 10 sccm argon flow rates, on both sides, using different deposition times, to transform them into conductive electrodes.

The glass/ITO pre-patterned substrate was cleaned with Hellmanex 2% solution, acetone, and isopropyl alcohol followed by plasma treatment for 15 min, before use. The PEDOT-PSS hole transport layer was deposited by spin-coating at 5000 rpm for 30 s and the CBP: (20 wt%) IrQ(ppy)_2_ emissive compound dissolved in DCM anhydrous was deposited by spin-coating at 3000 rpm and 30 s inside a glovebox. In all cases, the thickness of the active layer was around 50 nm.

The metalized electrode is thermally transferred to ITO/PEDOT-PSS/ CBP: 20 wt% IrQ(ppy)_2_ sandwich structure as a transparent cathode ensuring a double-side emission, further called OLED with electrospun cathodes. For comparison, another batch with the same deposition procedure for the ITO/PEDOT-PSS/CBP: 20 wt% IrQ(ppy)_2_ sandwich structure was achieved followed by the gold thin film deposition by magnetron sputtering at 18 mA and 10 sccm argon flow rate, further called OLED with thin film cathode.

### 2.3. Device Characterization

The obtained transparent cathodes were investigated through the optical transmittance using a Cary 5000 spectrometer, electrical conductivity using a four-point probe Ossila device, and morphological analysis by field emission scanning electron microscopy (FESEM) (Carl Zeiss–Gemini 500). For FESEM imaging, the double-sided gold-metalized nanofiber web was frozen in liquid nitrogen and then broken to ensure the cross-section of the electrospun cathodes.

The obtained OLED structures were characterized using current-voltage, capacitance-voltage, and luminance measurements. The current-voltage measurements were performed using a Keithley 2450 source meter, while the luminance was measured using a Konica Minolta CS-2000 spectroradiometer. For dielectric measurements, performed at room temperature, a UC2878 precision LCR meter was used, connected to a Peaktech DC voltage source for the capacitance frequency and capacitance-voltage techniques.

## 3. Results

### 3.1. Optical, Structural, and Electrical Measurements of the As-Prepared Transparent Electrospun Cathodes

**Figure 2:**
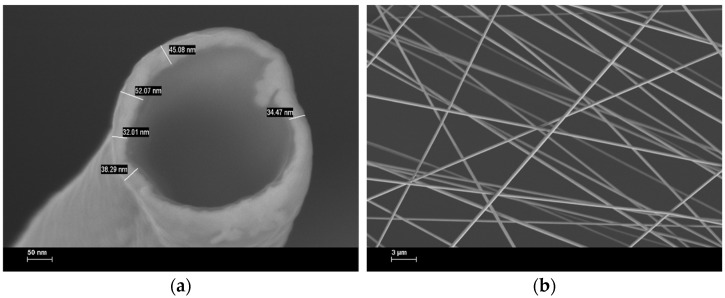
SEM images of electrospun gold cathodes (**a**) Cross-section at 300 kX; (**b**) Planar view at 5 kX.

The morphology of the obtained electrospun cathodes was analyzed using the SEM technique and, from the SEM images, the thickness of the gold layer deposited on the electrospun fiber webs was estimated. This is an important parameter, especially for the current-voltage characteristics and optical properties. The cross-section image (Figure 2a) shows the gold thickness of the frozen and broken PMMA/gold nanofibers.

The gold layer homogeneously covers the entire surface of the PMMA fiber, having approx. 40–50 nm thickness, which ensures a good charge injection from these electrospun cathodes. The area of these cathodes was estimated at 48 mm^2^, whereas the total thickness ranges between 240 nm and 350 nm. In a planar view and large area (Figure 2b), the SEM images demonstrate the uniform deposition of the gold layer and the overlapping between the nanofibers, which suppress the wire-to-wire resistance and the leakage current.

**Figure 3:**
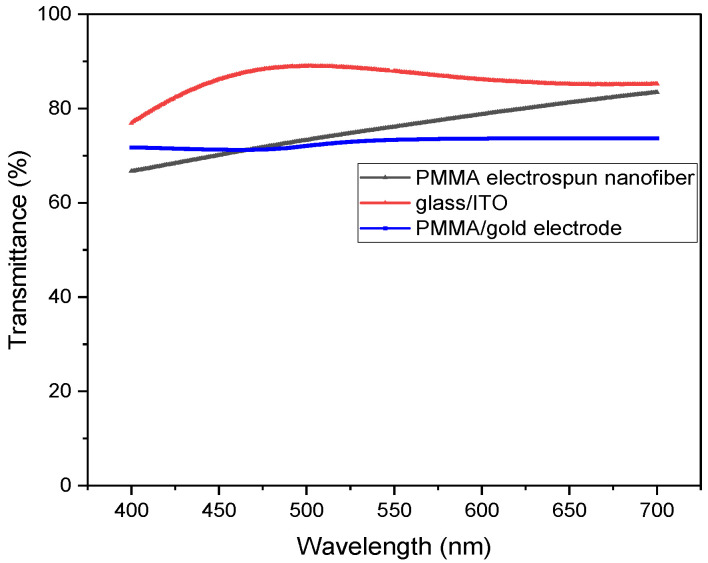
Optical transparency of the PMMA/gold cathodes.

The obtained TCE electrodes, as gold-covered PMMA templates, were thermally transferred firstly on the glass substrates for optical, electrical, and structural characterization. Optical transmission properties of the PMMA/gold cathodes were compared with the PMMA freestanding electrospun web and classical glass/ITO substrate as it is illustrated in Figure 3.

The transmittance in the electroluminescence range (500–700 nm) varies from 70% to 80% in the case of PMMA freestanding structure and PMMA/gold electrode, comparable with glass/ITO electrode. These values are in accordance with the electroluminescence properties which will be revealed in the next section. One can argue that after the sputtering deposition the gold film does not affect the transparency of the electrode web due to the proper optimization of the gold thickness and the higher transparency of the ITO anode will influence the emission properties of the bottom side of the OLED.

Direct Current (DC) electrical measurements of the obtained PMMA/gold nanofibers revealed low surface resistivity, making them suitable for the charge injection into the sandwich structure of the obtained OLEDs (Figure S1). The slopes of the current-voltage curves range from 1.62 to 1.84, revealing a sheet resistance in the PMMA/gold from 7 to 8.15 Ω/sq. For a 40–50 nm gold film deposited on the 300 nm PMMA nanofibers, the resistivity is 350 nΩm and 407 nΩm.

Concerning the transparency versus sheet resistance, in the case of the PMMA/gold nanoweb electrode, the optical transmission is higher (72% at 500 nm) compared with the thin gold layer (47% at 500 nm) for the same sheet resistance (Figure S2).

Figure S3 reveals the SEM images of the PMMA/gold nanofibers compared with PMMA/Al nanowebs in a tilted view (a,c) and a planar view (b,d). As we can see, in the case of PMMA/gold nanofibers it is a good connection between the nanofiber and a small roughness after the substrate attachment.

### 3.2. Current-Voltage Characteristics

**Figure 4:**
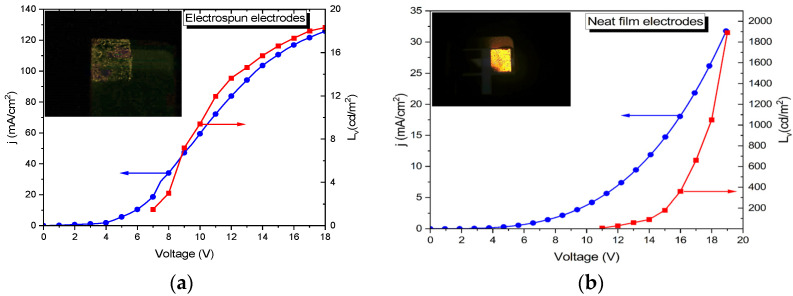
Luminance-current-voltage characteristics for an OLED with (**a**) an electrospun cathode; (**b**) a thin film cathode.

The presence of the electrospun electrodes enables a double-side emission compared with the single-face emission in the case of the classical OLED. Differences appear in the electrical properties and electroluminescence of those devices. Figure 4a reveals the luminance-current-voltage (L-I-V) characteristics of the OLED with electrospun cathodes, compared with the similar luminance-current-voltages of the OLED neat film cathodes (Figure 4b).

As can be seen, the current density of the device with cathodes obtained using the electrospun technique is about four times higher than that in the neat film device, while the luminance is a hundred times lower. This current density obtained for an electrode area of 0.48 cm^2^, is valid just for the gold neat film. In the case of the electrospun electrodes, the effective area is four times lower than in the case of the neat film, due to the free space between the nanofibers. The transmittance of these nanowebs is around 72−75%, meaning that just 25% of light is reflected by the fibers, reducing the area of the electrodes. Meanwhile, the luminance is considerably reduced, marking a different charge injection between these two devices due to different electrical contact properties.

**Figure 5:**
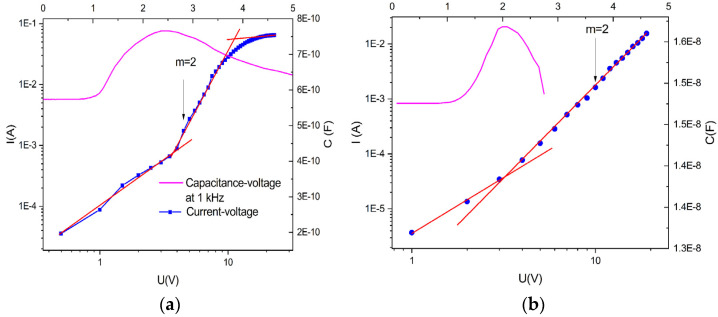
Capacitance–voltage and current–voltage curves in log–log scale of the: (**a**) electrospun devices; (**b**) neat film devices.

In the log-log representations, the current-voltage characteristics are also different, especially for medium and high voltages. At lower electric fields, both devices exhibit an ohmic characteristic, up to 2.5 V in the case of the electrospun cathode device (Figure 5a) and 2 V for the device with thin film electrodes (Figure 5b). Moreover, the current-voltage for the electrospun cathode devices exhibits a fast increase in the current with the applied voltage from 2.5 V to 10 V, followed by a roll-off region in which the current tends to saturate with the increasing voltage. In both cases, in the middle zone, the current-voltage dependence is given by the exponential law:

(1) $$J\sim V^{m}$$

where m = T_t_/T with T as the absolute temperature and T_t_ as the characteristic temperature of the trap distribution [24].

This dependence defines the diode character of the OLED devices, establishing the open voltages at the crossing point between the low and middle voltage domains. The OLED devices with neat film cathodes exhibit the same exponential law but with prominent curvature, extending the diode characteristics of these devices over a wider range of applied voltages.

At higher voltages, the OLED with electrospun cathodes, presents a saturation effect, meaning a higher trapping effect on either shallow or deep levels, which effectively reduces the electroluminescent effects [25]. This region is also known as a volume-controlled current with partly filled traps [26].

To underline the change point between leakage or diffusion-limited current caused by ohmic contact (low voltage region) and volume-controlled current with an exponential distribution of traps, known as the opening voltage of diodes, the capacitance-voltage measurements have been performed (Figure 5a,b). Recently, capacitance-voltage spectroscopy was used to investigate the physical mechanisms of OLEDs, where the first inflection point corresponds to the turn-on voltage [27]. In the case of the devices with electrospun cathodes, the inflection point was measured at 2.5 V while in the case of the neat film electrodes at around 2 V.

### 3.3. Electroluminescence Properties

**Figure 6:**
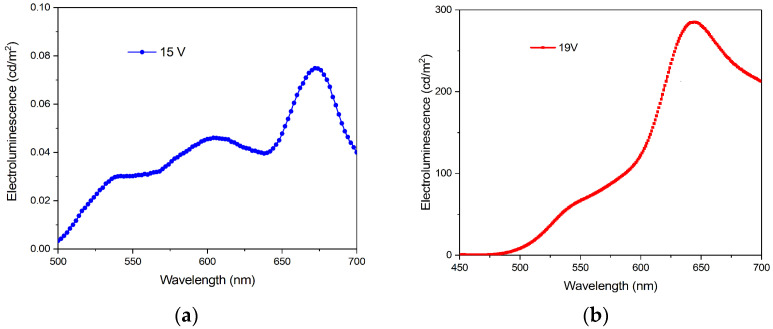
Electroluminescence of an OLED with (**a**) an electrospun cathode; (**b**) a thin film cathode.

Figure 6a shows the electroluminescence of the OLED with an electrospun cathode device at a stable point of functionality, 15 V and 55 mA, in which the emission spectrum has three peaks centered at 535 nm, 607 nm, and 670 nm, with intense red electroluminescence. The ratio between the green electroluminescence (535 nm) and the red one (670 nm) is around 2.5.

For the thin film device, the electroluminescence was measured at a stable point of functionality, 19 V and 16 mA, the emission spectrum has two main peaks, a green one at 535 nm and a large red one centered at 640 nm. The ratio between the green electroluminescence and the red one is around 4.5 (Figure 6b) [28].

It is important to mention two aspects: the first one is connected with the intensity of the electroluminescence, in which the light emitted from the neat film cathode device is four orders of magnitude higher than the similar electrospun cathode devices. The second one is related to the level of the current for the same area of electrodes, which is higher in the case of electrospun cathode devices, even for a lower applied voltage.

**Figure 7:**
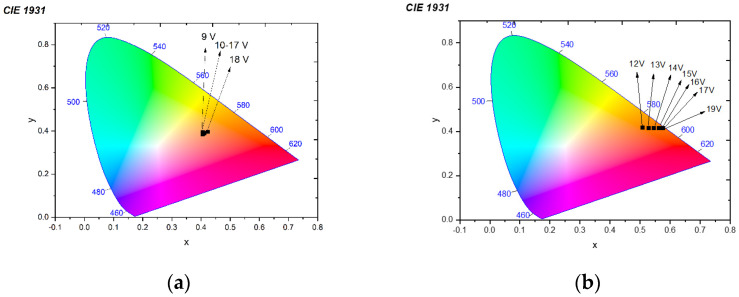
Electroluminescence versus applied voltage for the (**a**) electrospun cathode devices; (**b**) thin film cathode devices.

Concerning the dependence of the electroluminescence versus the applied voltage, the CIE representations of the two devices are given in Figure 7a,b.

The presence of the 607 nm electroluminescent peak in the case of the electrospun cathode devices and the ratio between the green and the red emissions, move the luminance in the CIE representation from the red zone (x = 0.5747, y = 0.4142) to the white region (x = 0.4078, y = 0.3872).

**Figure 8:**
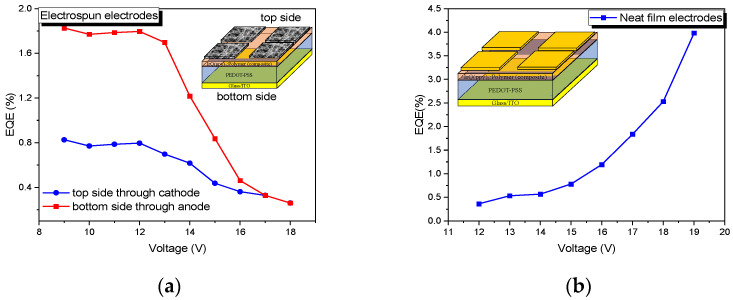
EQE versus the applied voltage for the (**a**) electrospun cathode and (**b**) the neat film cathode.

The external quantum efficiency was measured on both faces, where the top side defines the electroluminescence measured through the electrospun cathode, while the bottom side reveals the electroluminescence versus applied voltage through the glass/ITO substrate (Figure 8a).

The EQE versus the applied voltage for the electrospun cathodes is almost constant between 9 and 12 V, then decreases, confirming the roll-off region. However, the EQE is higher here around 0.8 % compared with that measured at 15 V. For the neat film cathodes, the EQE has an increased value up to an applied voltage of 19 V, where the EQE is around 4.2% (Figure 8b).

### 3.4. Dielectric Measurements

**Figure 9:**
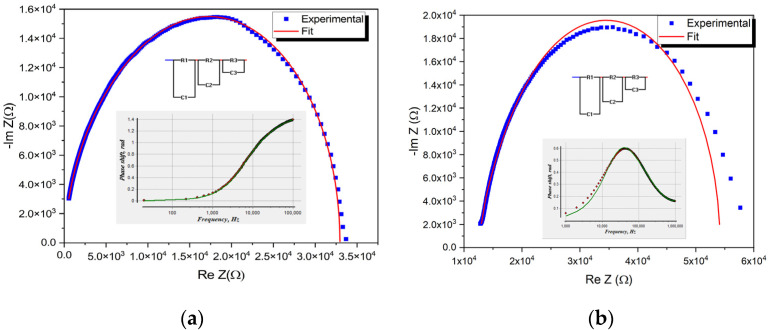
Measured Cole–Cole plots and the fitting for the devices with (**a**) an electrospun cathode; (**b**) a neat film cathode.

Capacitance-frequency measurements were performed to explain the simultaneous injection of holes from the ITO anode and electrons from the electrospun gold cathode. Figure 9a,b reveal the measured Cole-Cole plots together with the modeled equivalent circuit formed from three RC parallel contributions from each main layer for the OLED with electrospun and neat film cathode.

As can be seen, the phase shift versus frequency suggests a good fit for a model in both cases with three RC parallel circuits, and the fitting parameters are presented in Table 1. The R_1_-C_1_ accounts for the influence of the electrospun gold cathode, R_2_-C_2_ is connected with the charge transport in the active layer given by the CBP: 20 wt% IrQ(ppy)_2,_ and the last one, R_3_-C_3_ marks the influence of the anode, mainly given by the ITO/PEDOT: PSS contribution.

The fitting of the experimental curve provides more detailed information about the properties of structures and the results are given in Table 1. Both figures show that for the structures under study, the Cole-Cole plots are close to the semicircle for a single relaxation process because the above equation can be written as:

(2) $${\left[Re\;Z-\left(R_{S}-\frac{R_{p}}{2}\right)\right]}^{2}+{\left[Im\;Z\right]}^{2}=\frac{R_{p}{}^{2}}{2}$$

The semicircles are slightly deformed, which indicates the use of a more complex equivalent circuit with several relaxation times. The obtained results were compared with similar device structures [29].

## 4. Discussion

Optical, structural, and electrical measurements of the as-prepared transparent electrodes have shown good transparency and higher electrical conductivity.

The electrical conductivity of the metallic electrodes is influenced by the large wire–wire junction resistance between the metallic nanowires, which in some cases could exceed one kΩ/sq or even one GΩ/sq and influences the charge injection in the OLED structures [30]. The most common method for the reduction in the wire-wire resistance is the welding process obtained using different techniques such as thermal annealing, electrochemical deposition of gold over the silver nanowires [31], which reduces the sheet resistance below 100 Ω/sq, plasmonic welding [32] or cold pressing [33], which decreases the sheet resistance up to 8.6 Ω/sq. Moreover, the optical transmittances in the case of silver nanowires vary from 89% to 94%. For the gold nanowires, the sheet resistance varies from 400 Ω/sq for the transparent electrodes with a high 96% transmittance [34] to 49 Ω/sq and 83% transmittance [35]. In our case, the electrical conductivity is better than other gold nanowires and even better than the similar silver nanowires, up to 7–8.15 Ω/sq, but for a lower transmittance, which varies from 70 to 75% (at 500 nm) for the PMMA/gold electrospun cathodes.

The presence of the hole injection ITO/PEDOT: PSS structure leads to a double-injection device with electroluminescent properties [36]. Besides the space-charge limited effects, the electron-hole recombination processes are effective, creating an interplay between the charge injection and the recombination processes. In the case of the neat film electrodes, the ohmic behavior of the J-V curves at low voltages is extended to 2 V, while in the case of electrospun gold cathodes, this zone is almost the same at about 2.5 V [37]. For the medium voltages, up to 10 V, the current density–voltage in the OLEDs based on the neat film electrodes is governed by a high-power law. This region is known as the trapped-charge zone, which limits the charge transport in the bulk [25].

In the case of PMMA/gold electrospun cathodes, the power law gives m-values around two or lower, indicating a combined process between space-charge limited and the recombination effects, which enhances the electroluminescence processes. In the high-voltage zone, from 10 V to 18 V, the curve shows a slight decrease in the current density together with a decrease in the electroluminescence intensity. This is known as a roll-off process, a rectification process, which can be attributed to the lack of the charge balance or exciton quenching. In the electro-optical model of the OLED, this effect is assigned to a non-emitting diode with a lower emission coefficient than the emissive diode, which acts from 2.5 to 10 V [38]. This effect is related to the non-uniform charge injection from the electrospun PMMA/gold cathodes, which create a non-uniform electric field around their circular structure.

Capacitance-voltage measurements confirm the turn-on voltage point between the ohmic zone and the zone dominated by the exponential distribution of the traps. This point marks a constant injection in the devices when the OLED starts to be driven by the applied voltage, increasing the capacitance [27].

Concerning the electroluminescence of the IrQ(ppy)_2_ based devices with neat film cathodes versus the emitted wavelengths, the emission consists of three peaks one around 535 nm given by the metal-to-ligand charge transfer (MLCT) from the Ir^3+^ toward the phenylpyridine ligand and one at 640 nm given by the metal-to-ligand charge transfer from the Ir^3+^ toward the quinoline ligand [22]. The all-over color is orange and appears as a mean value from 580 to 590 nm. The red one was higher than the green one due to the different charge transfer under various current densities. The red shift of the electroluminescence in the neat film cathode devices with the applied voltage reveals a dependence of the MLCT processes from the phenylpyridine to the quinoline ligand, influenced by the lower current density. It seems that a higher current density favored the metal-to-ligand charge transfer (MLCT) from the Ir^3+^ toward the phenylpyridine ligand, while a lower current density favored the metal-to-ligand charge transfer (MLCT) from the Ir^3+^ toward the quinoline ligand.

In the electrospun cathode devices, the third electroluminescent peak at 607 nm is probably due to the electroluminescence from the triplet states in the CBP polymers [39]. This peak could appear as triplet-triplet interaction between the triplet states from IrQ(ppy)_2_ responsible for the green emission with the triplet states from CBP [40].

The external quantum efficiency of the electrospun cathodes, measured on both sides, reveals the double side emission, but the efficiencies are different between the top and bottom sides, as can be seen in the graphical abstract. The bottom side is more efficient due to the lack of a shadowing process, which appears when the light is measured from the top side. This fact is given by the 25% of light is reflected by the fibers, which reduces the area of the electrodes. The bottom side is favored also by the better charge injection in these OLED structures based on the TCE electrodes.

The dielectric measurements confirm the double-charge injection mechanism in which the assignment of the electrospun device with three parallel circuits describes the processes at the cathode, in the active layer and anode, ignoring the presence of the PEDOT: PSS layer [29].

The main difference between the electrospun cathodes and neat films is given by the lower resistance R1 in the first case, which underlines a non-uniform injection of the electrons from the electrospun cathode compared with the one from the neat films.

For a single charge injection of PMMA/Au/Alq3/LiF/Al, in which all layers are *n*-type, the cathode contribution given by the R1-C1 parallel circuit has similar values with R1=1.71 kΩ and C1=8.6 nF [41].

## 5. Conclusions

PMMA/gold-covered nanofibers can be efficiently used as cathodes for OLEDs enabling the construction of double-side electroluminescence devices. These cathode nanofibers obtained by electrospinning technique using the top-down method, enable the double-side emissions of these devices and can be used for the production of foldable electroluminescent devices. The geometry and roughness of the obtained transparent electrodes change the contact resistance at the interface with the electroluminescent layer, but the values concerning the depletion, which are connected with the capacitances (approximative the same values), obtained from the anode and cathode are similar to the electrodes obtained as thin films. This fact confirms a non-uniform charge injection from the electrospun cathodes, which changes the electroluminescence properties of these devices, due to low contact resistance between the cathode and the active layer, leading to a balance between the hole and electron concentrations, which facilitates the direct appearance of the trapped-charge zone.

The fitting procedure with three RC parallel circuits gives better results in the case of double-charge transport devices and underlines the lower contact resistance between the electrospun cathode and the active layer.

At higher voltages, the obtained electroluminescence shows a roll-off region where the electroluminescence tends to a saturation process with the injected current. In this case, the domain for the applied voltages increases the electroluminescence is restrained between 2.5 V and 10 V, sustaining the non-uniform injection of the electrons in the electrospun devices.

The use of these transparent nanofiber electrodes enables a better charge injection in the OLED structure, confirmed by the electroluminescence of these devices and leading to a further step toward the flexible OLEDs.

## Figures and Tables

**Table 1:** Parameters of the equivalent circuit for the electrospun and neat film cathodes.

Assignments	Nanoweb	Errors %	Ref [29]	Film	Errors %
Cathode	R_1_ = 278 Ω C_1_ = 3.66 nF	0.71 1.53	R_1_ = 1.51 kΩ C_1_ = 8.9 nF	R_1_ = 1.3 kΩ C_1_ = 9.36 nF	0.07 1.59
Active layer 10 kHz−100 kHz	R_2_ = 3.85 kΩ C_2_ = 1.71 nF	0.24 0.13	R_2_ = 33 kΩ C_2_ = 4 nF	R_2_ = 30.08 kΩ C_2_ = 4.21 nF	1.66 1.22
Anode 20 Hz−10 kHz	R_3_ = 28.82 kΩ C_3_ = 0.78 nF	0.11 0.05	R_3_ = 391 kΩ C_3_ = 4 nF	R_3_ = 38.17 kΩ C_3_ = 2.16 nF	0.65 0.34

## Supplementary Materials

The following supporting information can be downloaded at: https://www.mdpi.com/article/10.3390/mi14030543/s1, Figure S1: Current-voltage dependence of the gold electrospun cathodes; Figure S2: Comparison between the transmittances of the PMMA/gold electrospun and neat film with the same sheet resistance; Figure S3: SEM images of the sample covered with gold (a); (b) aluminum (c); (d) on both sides and attached to the glass substrate.
