# Supplementary material for: Organic Light-Emitting Diodes with Electrospun Electrodes for Double-Side Emissions
Source: Micromachines (Basel). 2023 Feb 25;14(3):543. doi: 10.3390/mi14030543 (PMC10056642; doi:10.3390/mi14030543)
Supplement: Supplementary file 1 [file micromachines-14-00543-s001.zip › micromachines-2209852-supplementary.pdf]

micromachines

MDPI

Supplementary information

Organic Light-Emitting Diodes with Electrospun Electrodes for Double-Side Emissions

Iulia Corina Ciobotaru, Monica Enculescu, Silviu Polosan, Ionut Enculescu and Constantin Claudiu Ciobotaru *

National Institute of Materials Physics, Laboratory of Functional Nanostructures, Atomistilor 405A, 077125 Magurele, Romania; corina.ciobotaru@infim.ro (I.C.C.); mdatcu@infim.ro (M.E.); silv@infim.ro (S.P.); encu@infim.ro (I.E.)

* Correspondence: claudiu.ciobotaru@infim.ro

Sheet resistance measurements.

DC electrical measurements of the obtained PMMA/gold nanofibers revealed low surface resistivity, making them suitable for the charge injection into the sandwich structure of the obtained OLEDs (figure S1).

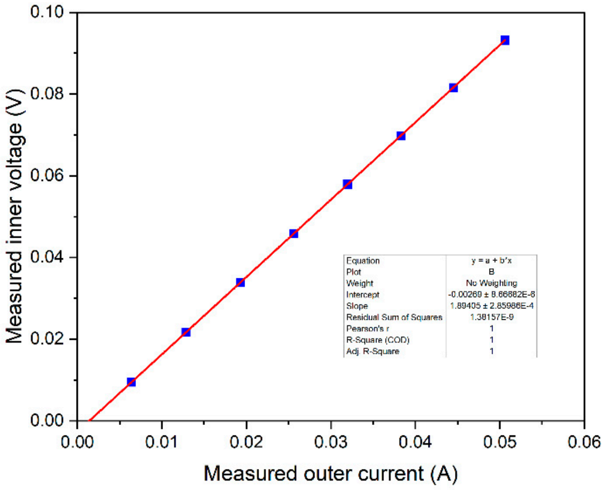

Figure S1. Current-voltage dependence of the gold electrospun cathodes.

The four-point probe measurements on the PMMA/gold fibers suggest a good sheet resistance, from 7-8.15 Ω/sq depending on the area of measurements, because in the center of the sample the sheet resistance is higher due to the non-uniform gold covering.

This fact leads to a resistivity between 350 nΩ*m and 407 nΩ*m., based on the following relation:

$$\rho = R_s * t \qquad [\Omega\text{*m}] \tag{1}$$

where t-is the thickness of the sample, around 40-50 nm and R_s is the sheet resistance given by:

$$R_s = \frac{\pi}{\ln 2} * \frac{\Delta V}{\Delta I} * C \qquad [\Omega/\text{sq}] \tag{2}$$

The above equation accounts for the slope between the inner voltage and the applied current on the inner electrodes of the four-point probe equipment. Here, C is a correction factor for a square sample of 6x8 mm^2, with a value of 0.95.

To underline the superior properties of the electrospun electrodes versus metallic thin films, a thin layer of gold with the same sheet resistance as PMMA/gold was prepared. In the case of PMMA/gold web electrode, the optical transmission is higher (72% at 500 nm) compared with a thin gold layer (47% at 500 nm).

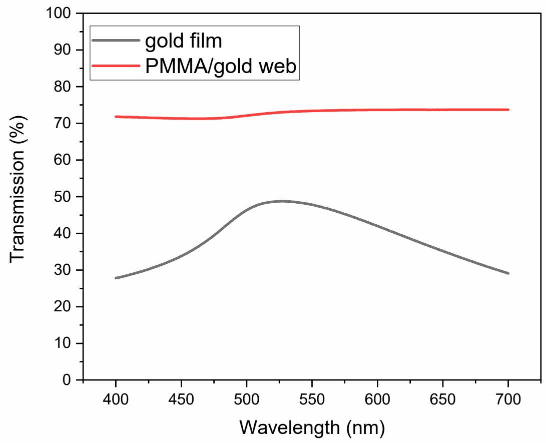

Figure S2. Comparison between the transmittances of the PMMA/gold electrospun and neat film with the same sheet resistance.

The transparent conductive electrodes obtained as thin films should fulfill two conditions:

High transparency

High electrically conductivity

The decrease in the metal thickness increases the transparency and drastically increases the sheet resistance when the metal thickness is comparable or lower with the mean free path of the electrons in the metal, which is 40 nm in gold. The bump in the figure is due to the scattering on the gold nanoparticles as a Mie effect.

Gold has a higher work function and the most suitable materials for cathodes are Ag or Al due to their lower work function. However, for the metallic covering of nanofibers, the gold shell adheres very well to the ITO/PEDOT-PSS/ CBP: Irq(ppy)$_2$ (20%) sandwich structure, compared with the silver or aluminum, after the thermal transfer. For Ag NW networks, a considerable part of NWs is not strongly adherent to the substrate, thus the suspending NWs are easy to detach. In contrast, metal interconnects made using top-down methods like Au nano-meshes are more adhesive to the substrate. To demonstrate the adhesion of the PMMA gold-covered nanofibers, two samples of PMMA/gold and PMMA/aluminum with the same optical transmission were prepared and attached to the nanowebs on a glass substrate. Afterward, SEM analysis was performed in a tilled view (a, c) and a planar view (b, d) (figure S3).

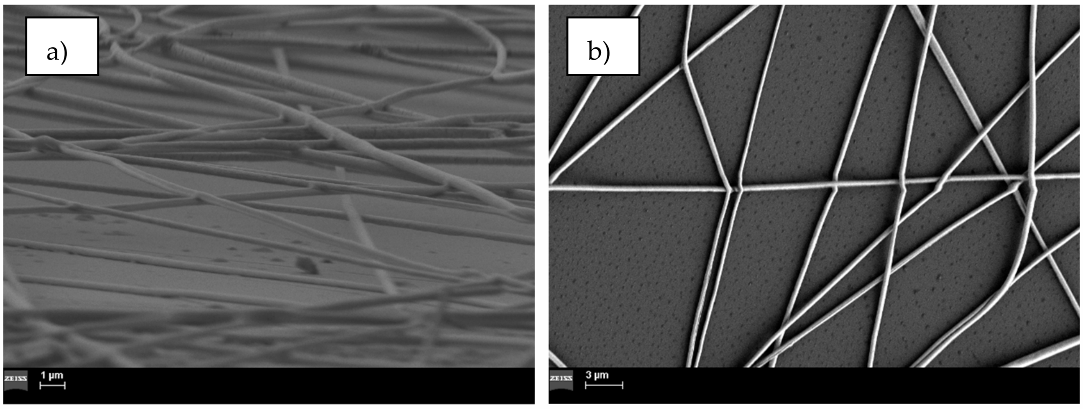

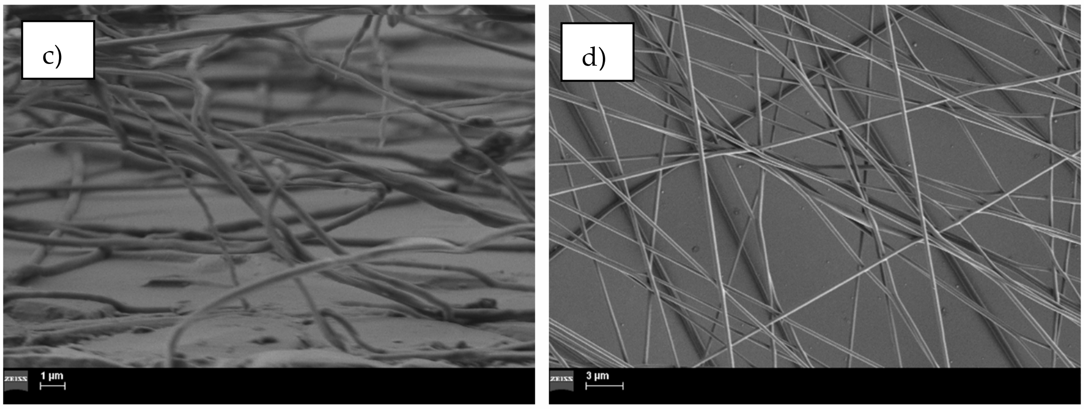

Figure S3. SEM images of the sample covered with gold a); b) aluminum c); d) on both sides and attached to the glass substrate.

In the case of PMMA/gold nanofibers, a good connection between the nanofiber and a small roughness after the attachment on the substrate, compared with the PMMA /Al nanowebs is observed. This fact was also observed in the case of PVA nanofiber templates covered with copper, silver, and aluminum [16].

Moreover, gold does not have any oxidation problem, compared with silver or aluminum, and is therefore chemically stable in the air [19]. For instance, the air non-stable metals involve supplementary steps like the encapsulation of the final device, which could lead to cathode fiber breakage.
